# Reproductive Failure in Smallholder Pig Farms in East and Southeast Asia: A Systematic Review

**DOI:** 10.3390/ani15091226

**Published:** 2025-04-26

**Authors:** Belete Haile, Esa Karalliu, Jeremy Ho, Karyn A. Havas, Renata Ivanek, Joyce Ip, Chen Xin, Omid Nekouei

**Affiliations:** 1 Department of Infectious Diseases and Public Health, Jockey Club College of Veterinary Medicine and Life Sciences, City University of Hong Kong, Hong Kong SAR, China; ekaralliu2-c@my.cityu.edu.hk (E.K.); milaxin2-c@my.cityu.edu.hk (C.X.); omid.nekouei@cityu.edu.hk (O.N.); 2 Centre for Applied One Health Research and Policy Advice (OHRP), City University of Hong Kong, Hong Kong SAR, China; joyceip@cityu.edu.hk; 3 Agriculture, Fisheries and Conservation Department, Government of the Hong Kong SAR, Hong Kong SAR, China; 4 Department of Public and Ecosystem Health, College of Veterinary Medicine, Cornell University, Ithaca, NY 14853, USA; 5 Department of Population Medicine and Diagnostic Sciences, College of Veterinary Medicine, Cornell University, Ithaca, NY 14853, USA; ri25@cornell.edu

**Keywords:** reproduction, infectious disease, smallholder farming, swine, Asia

## Abstract

Reproductive failure has significant socioeconomic impacts on smallholder pig farms. This systematic review was conducted to compile the types of reproductive failures and their underlying causes reported in smallholder pig farms from East and Southeast Asia and to identify relevant knowledge gaps. Following the Preferred Reporting Items for Systematic Reviews and Meta-Analyses (PRISMA) guidelines, 26 peer-reviewed studies met the eligibility criteria and were included in our synthesis. These studies were conducted in 11 countries, with Vietnam, China, and Thailand representing the highest share (53.8%). Only six studies (23%) investigated reproductive failure as their primary objective. Stillbirth, mummification, late-term abortion, and weak-born piglets were the predominant reproductive failures reported from smallholder pig farms across the region. The most frequently cited viral pathogens associated with these failures were porcine reproductive and respiratory syndrome virus (PRRSV) and porcine circovirus type 2 (PCV-2). Common non-infectious risk factors included extreme climate conditions (e.g., heat stress), poor diet and housing, and suboptimal boar management. Our synthesis highlighted a dearth of research focused on reproductive failure in smallholder pig farms in the region and emphasised the need for more targeted studies to clarify the biological, environmental, and managerial risk factors contributing to reproductive failure. This will facilitate the development of targeted prevention and control measures that account for the unique farming conditions and challenges smallholder farms face in East and Southeast Asia.

## Simple Summary

Reproductive failure is the primary cause of culling breeding sows in commercial farms, which can manifest in various forms, such as anoestrus, abortion, stillbirth, and conception failure. Many risk factors, including infectious diseases, can contribute to reproductive failure in pig herds, with particular concerns in East and Southeast Asia due to specific environmental conditions (e.g., high temperature and humidity). We conducted the current systematic review to compile the types of reproductive failures and their underlying causes reported in smallholder pig farms in East and Southeast Asia and identify relevant knowledge gaps. Of 26 eligible studies in our synthesis, only 6 investigated reproductive failure as their primary objective. Stillbirth, mummification, late-term abortion, and weak-born piglets were the predominant reproductive failures reported from smallholder pig farms across the region. The most frequently cited viral pathogens associated with these failures were porcine reproductive and respiratory syndrome virus and porcine circovirus type 2. Common non-infectious risk factors included extreme climate conditions (e.g., heat stress), poor diet and housing, and suboptimal boar management. Our findings lay the foundation for future research and facilitate the development of targeted prevention and control measures that account for the unique farming conditions and challenges in East and Southeast Asia.

## 1. Introduction

Reproductive failure is the primary cause of culling breeding sows in commercial farms [1,2,3]. In Southwest China, an analysis of 19,471 culling records from 24 swine herds indicated that reproductive failure, including nonpregnancy, return to oestrus, abortion, and prolonged anoestrus, accounted for approximately 30% of total culling decisions [4]. Similarly, a study of 4887 culled sows in Thailand found old age (34.9%) and reproductive disorders as the primary reasons for culling [5]. Reproductive failure can manifest in various forms, such as anoestrus, abortion, stillbirth, conception failure, pseudopregnancy, and other complications occurring during the pig reproductive cycle [6,7]. Many risk factors, including infectious diseases, inadequate management practices, environmental stress, nutritional deficiencies, and genetic factors, can contribute to reproductive failure in pig herds [8].

Pig reproductive failure is of particular concern in East and Southeast Asia due to environmental factors characterised by high temperatures and humidity, which lead to reduced farrowing rates and seasonal infertility [9,10,11,12]. Southeast Asian countries and some provinces in China are vulnerable to climate hazards, including tropical cyclones and heat waves, which contribute to the collective stress level in breeding pigs, particularly during the summer months [13,14]. For instance, a study conducted in Southern China reported a high proportion of anoestrus cases (47.7%) among 4534 sows across four farms, with the highest occurring in June (11.8%) and July (17.7%) [1]. In addition to climate stressors, the widespread infectious diseases among domestic and wild pig populations severely impact reproductive performance [15,16,17]. Porcine reproductive and respiratory syndrome (PRRS), for example, has caused substantial economic loss in breeding herds across the region due to many cases of abortion, stillbirth, and weak piglets over the years [18,19]. Japanese encephalitis virus (JEV) is also a significant concern for the swine industry in the region, as it causes adverse reproductive effects, including abortion, mummified foetuses, and stillbirth in sows, orchitis, and a decline in semen quality in boars [20].

Pig-rearing systems in Asia exhibit considerable diversity, ranging from small units of scavenging and backyard operations to large-scale intensive enterprises [21,22]. Among these, smallholder pig farms are particularly dominant in Southeast Asia, where they play a critical role in rural livelihoods and food security [23]. These farms are integral to the socioeconomic fabric of rural communities, providing household income, serving as a primary source of animal protein, supplying manure for crop production, and offering a sustainable means of livelihood for millions of households [22]. However, smallholder pig farms face numerous challenges that limit their productivity. These challenges include reliance on indigenous breeds with low genetic potential, limited veterinary services, and suboptimal feeding practices, resulting in poor reproductive performance and slower growth rate [10,24,25]. Enhancing the productivity of these systems is imperative to strengthening their contribution to rural livelihoods and regional food security [26].

Reproductive performance is a key determinant of the productivity and profitability of pig production systems. Nevertheless, reproductive failure remains a persistent constraint, undermining the long-term sustainability of smallholder farms [24]. Addressing this issue necessitates a comprehensive understanding of the underlying causes of reproductive failure and the implementation of evidence-based, targeted interventions. Research efforts in extensive production systems for pigs and other livestock species have shown promising outcomes in improving reproductive parameters [27]. Integrating data-driven herd management strategies enables veterinarians and pig producers to optimise reproductive efficiency and overall system productivity [28].

While health and production management practices in intensive farms are well documented [28], data from smallholder pig farms in East and Southeast Asia remain scarce and fragmented. This lack of information and inconclusive evidence limits veterinarians, para-veterinarians, and other stakeholders from effectively supporting farmers to improve their production capacities.

Recent research highlights the challenges and opportunities in smallholder pig farming across East and Southeast Asia. While historically understudied, these production systems have recently gained significant attention following African swine fever (ASF) outbreaks due to their perceived role in virus transmission dynamics [29,30]. While much of the recent research has concentrated on ASF control and containment, there remains a clear paucity of evidence and practical recommendations to address basic health and production management issues within smallholder systems [31]. Therefore, this systematic review was conducted to compile the types of reproductive failure and their underlying causes and risk factors reported from smallholder pig farms in East and Southeast Asia and to identify relevant knowledge gaps.

## 2. Materials and Methods

### 2.1. Search Strategy

This systematic review was conducted according to the Preferred Reporting Items for Systematic Reviews and Meta-Analyses (PRISMA) guidelines [32]. Our research question was “what are the reported types, risk factors, and causes of pig reproductive failure in the smallholder farms of East and Southeast Asia?” Five electronic databases, including three in English (PubMed, CAB Abstracts, and Scopus) and two in Chinese (China National Knowledge Infrastructure (http://www.cnki.net/) and Wanfang (http://www.wanfangdata.com.cn/index.html) were searched to gather all published peer-reviewed studies on pig reproductive health and failure in the region. The search domains and terms are presented in Table 1 and Table A1. The search time frame was between 1 January 2000 and 9 August 2023.

### 2.2. Eligibility Criteria

Studies that met the following criteria were included in our final synthesis: (1) published in peer-reviewed journals, (2) full text available in English or Chinese, (3) investigated pig reproductive failures or associated factors in smallholder farms (including studies that either did not specify the farm scale or included both smallholder and large-scale farms, with special focus on smallholders), and (4) conducted in East or Southeast Asian countries (i.e., Brunei, Cambodia, China, Hong Kong SAR, Indonesia, Japan, Lao PDR, Macau, Malaysia, Mongolia, Myanmar, North Korea, the Philippines, Singapore, South Korea, Taiwan, Thailand, Timor-Leste, and Vietnam [33,34]. Studies were excluded if they (1) were conducted in medium- or large-scale production systems, (2) were conducted on species other than domesticated pigs (Sus scrofa domestcus), (3) were laboratory experiments, (4) reported reproductive performance in normal circumstances (i.e., no reproductive issues/failure reported), or (5) were review articles and grey literature.

### 2.3. Study Selection Process

**Figure 1:**
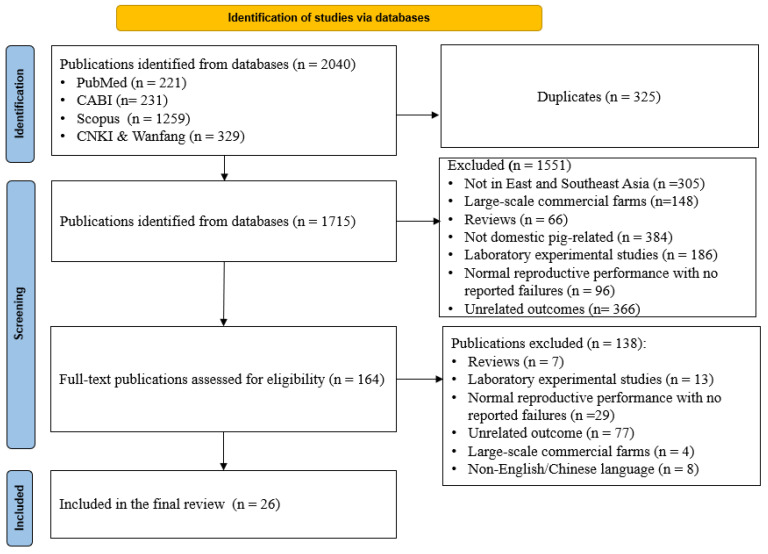
Preferred Reporting Items for Systematic Reviews and Meta-Analyses (PRISMA) flowchart, illustrating the screening and selection process of studies on pig reproductive failure in smallholder pig farms in East and Southeast Asia.

All steps of the study selection process are summarised in Figure 1. The initial search results were screened for the eligibility criteria in two steps. In the first step, all studies from the five databases were exported to EndNote v20 desktop software [35] to identify and remove duplicates. The remaining studies were uploaded to the free web tool Rayyan (http://rayyan.qcri.org) for title/abstract screening [36]. Two independent reviewers for each database (B.H. and E.K. for English and J.H. and J.I. for Chinese databases) screened the titles/abstracts of the retrieved studies using the outlined eligibility criteria. In the second step, eligible studies were carefully and fully assessed for eligibility by the same reviewers. Disagreements between the pairs of reviewers were resolved by a third reviewer.

### 2.4. Data Extraction and Synthesis

A custom-made table was created in Microsoft Excel 2016, and relevant information was extracted from the selected studies (Table 2) and qualitatively synthesised.

### 2.5. Quality Assessment of Included Studies

The quality of the included studies was evaluated using the guide notes in the *Cochrane Handbook for Systematic Reviews of Interventions* [37], which were adapted to our study types. This is a practical domain-based evaluation, including 10 main quality assessment domains (aim, study population, study power, masking, case definition, diagnostic classification, indication of precision, completeness of results, believable conclusions, and consistency). Critical appraisals were made separately for each domain. The risk of bias for each domain was defined as high, low, or unclear based on a detailed review of each selected study.

## 3. Results

Figure 1 illustrates the selection and screening process of the studies. The initial search across the five electronic databases identified 2040 records. After removing duplicates (*n* = 325), the titles/abstracts of 1715 records were screened, and 164 publications were deemed eligible for a full-text review. Of the 164 publications, 26 studies were selected for the final data extraction, while the remaining 138 publications were excluded due to unrelated study outcomes and the reporting of normal reproductive performance without any specific reproductive failure.

### 3.1. Characteristics of Included Studies

The key characteristics of the 26 included studies are summarised in Table 3. These studies were published between 2002 and 2022, with 54% (14/26) published after 2018. Twenty-four studies were published in English, and the remaining two were published in Chinese. These studies were sourced from 6 regional and 12 international journals. *Preventive Veterinary Medicine* and *Tropical Animal Health and Production* had the highest share, with five and four studies, respectively (Table A2). The studies were conducted in 11 countries, with the highest share from Vietnam (6/26) and four each from China and Thailand.

Of the 26 studies, 24 (92%) were observational (Table 3), and 2 had an experimental design [38,39]. Regarding study objectives, only 6 studies (23.1%) specifically targeted reproductive failure types and their underlying causes, while 13 studies (50%) focused on disease prevalence estimation and associated risk factors. One study assessed the financial impacts of PRRS, foot-and-mouth disease (FMD), and porcine epidemic diarrhoea (PED) in three provinces of Vietnam [40]. The remaining six studies (23.1%) examined the impacts of non-infectious factors on the reproductive performance of sows and boars.

Of the six studies targeting reproductive issues as their objectives, three field studies from China revealed frequent cases of endometritis and its role in causing infertility in sows on small-scale farms [41,42,43]. Major endometritis-associated risk factors reported by the authors included non-standard breeding practices, poor disinfection, birth complications, and infections with swine pathogens (e.g., swine fever, parvovirus, Japanese encephalitis, and swine pseudorabies). The remaining three laboratory-based studies investigated outbreaks characterised by increased rates of late-term abortions, stillbirth, mummified foetuses, and premature farrowing and provided some insights into potential causes. One study detected PCV-2 in weak-born piglets in Japan’s farrow-to-finish sow herd [44], and another study reported coinfection with PCV-2 and PRRSV in cases of stillborn piglets in Taiwan [45]. The third study reported various reproductive issues in sows naturally infected with a highly pathogenic PRRSV, including foetal death, premature birth, mastitis, agalactia, and oestrus disorders [46].

There were 13 studies targeting the prevalence of infectious diseases, and reproductive issues were not their primary focus. Nevertheless, diseases investigated in these studies are widely recognised as established causes or main risk factors for reproductive failure in swine production. Viral pathogens, particularly PRRSV and PCV-2, were the most frequently reported pathogens associated with stillbirth, mummification, weak piglet birth, and late-term abortion (Table 3).

Nine studies (35%) reported non-infectious factors, including the hot season, inadequate nutrition, improper housing, boar breed differences, and suboptimal boar management, which contributed to low reproductive performance and related reproductive issues in East and Southeast Asia. For example, an analysis of 711 ejaculates from 87 boars (including Duroc, Landrace, Yorkshire, and Landrace–Yorkshire-crossbred) demonstrated that Duroc boars had lower semen volume and total sperm production than other breeds during June and July (the hot season) in Thailand [38]. Another study on native Moo Lath pigs in Lao PDR revealed that providing nesting materials without ad libitum water access could potentially increase susceptibility to heat stress in sows [39]. More details on the 26 studies, including the authors’ practical recommendations, are presented in Supplementary Materials (Table S1).

**Table 3:** Summary of 26 studies on reproductive failure in smallholder pig farms across East and Southeast Asia.

Country	Reproductive Failure Manifestations	Infectious Agents *	Non-Infectious Factors	Study Type	Year Conducted (Study Period)	Ref.
Cambodia	Abortions, mummified foetuses, stillbirth, weak births	JEV		Cross-sectional	2019 (Oct.)	[47]
China	Pseudopregnancy, dystocia, stillbirth, weak births, and anoestrus	PPV, PRRSV, PCV-2, PRV, and JEV	Wrong use of drugs, poor semen quality, non-standard AI, same-boar use for years, poor nutrition, and extensive management	Case report	2020 (Oct.–Dec.)	[43]
China	Endometritis (infertility)	PPV, JEV, PRV, and PRRSV	Improper semen collection and improper assistance during parturition	Cohort	2012 (Jan.–Dec.)	[42]
China	Not specified	PRV		Cross-sectional	2013–2016	[48]
China	Infertility, decreased reproductive rate, stillbirth, and mummified foetuses	PRRSV, PRV, PPV, JEV, Brucella, and Chlamydia	Poor nutrition, natural breeding, poor ventilation, outdated housing, inadequate temperature control, and inappropriate use of drugs (e.g., dexamethasone)	Survey	Not specified	[41]
Hong Kong SAR, China	Not specified	PRRSV and PCV-2		Cross-sectional	2020 (Feb.–Mar. 2021)	[49]
Indonesia	Not specified	CSFV		Cross-sectional	2010 (Apr.–May)	[50]
Indonesia	Not specified	CSFV		Cross-sectional	2010 (Apr.–Sept.)	[51]
Japan	Mummified and late-term dead foetuses	PCV-2		Case report	2001 (Jun.–Aug.)	[44]
Lao PDR	Not specified	CSFV, erysipelas, FMDV, and PRRSV		Cross-sectional	2011 (NS)	[52]
Lao PDR	Stillbirth and remating		Lack of ad libitum water supply for gilts and sows and provision of nesting material without water supply	Interventional	2014 (Jul.–Dec. 2015)	[39]
Lao PDR	Infertility		Poor boar management and uncontrolled breeding	Cross-sectional	2009 (Feb.–Jun.)	[53]
Philippines	Not specified	PRRSV and PCV-2		Cross-sectional	NS	[54]
Taiwan	Late-term abortions, stillbirth, and premature farrowing	PCV2 and PRRSV		Case report	2016 (Nov.–Feb. 2017)	[45]
Thailand	Not specified		Hot season and boar breed differences	Intervention	2020 (May–Jul.)	[38]
Thailand	Infertility, stillbirth, return to oestrus, and mummified foetuses		Heat stress in sows	Retrospective	2018 (Aug.–Dec. 2020)	[55]
Thailand	Not specified	CSFV, PRRSV, PCV2, and IAV		Cross-sectional	2016 (Sep.–Feb. 2017)	[56]
Thailand	Not specified		Lack of boar stimulation and use of natural mating	Cohort	2005 (Jan.–Dec.)	[25]
Timor-Leste	Mummified foetuses and birth defects	CSFV		Cross-sectional	2013 (Mar.–Apr.)	[57]
Timor-Leste	Infertility		Uncontrolled breeding, natural breeding, and knowledge gap in detecting oestrus and gestation	Cross-sectional	2018 (Sep.–Dec.)	[58]
Vietnam	Not specified	*Leptospira* spp.		Cross-sectional	1998 (Apr.–May)	[59]
Vietnam	Abortion, neonatal death, and weak piglets	PRRSV		Cross-sectional	2002 (Jul.–Sep.)	[60]
Vietnam	Not specified	PRRSV, FMDV, and PEDV		Cross-sectional	2013 (May–Aug. 2014)	[40]
Vietnam	Foetal death and premature birth, mastitis, agalactia, and oestrus disorders	PRRSV		Case series	2010 (Not specified)	[46]
Vietnam	Not specified	PRRSV		Case–control	2010 (Apr.–Jun)	[61]
Vietnam	Not specified	PCV2, PRRSV, *M. hyopneumoniae,* JEV, and *Leptospira* spp.		Cross-sectional	Not specified	[16]

* Acronyms used in the table: AI (artificial insemination), CSFV (classical swine fever virus), FMDV (foot-and-mouth disease virus), IAV (influenza A virus), JEV (Japanese encephalitis virus), *M. hyopneumoniae* (*Mycoplasma hyopneumoniae*), PCV-2 (porcine circovirus type 2), PPV (porcine parvovirus), PRRSV (porcine reproductive and respiratory syndrome virus), PRV (pseudorabies virus).

### 3.2. Quality Assessment (Risk of Bias)

**Figure 2:**
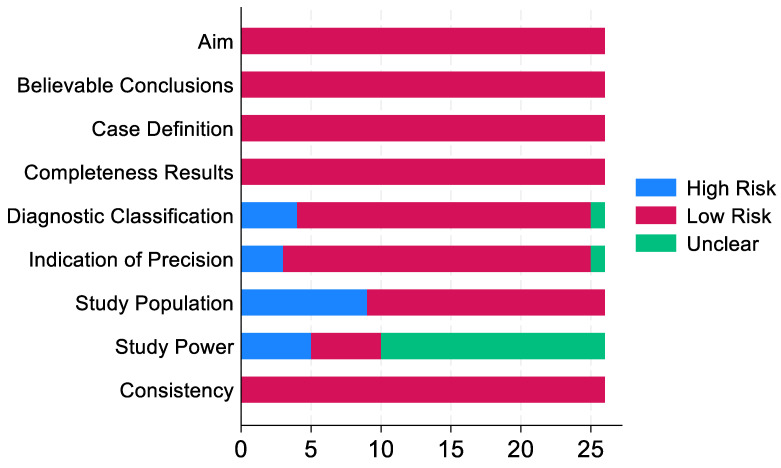
Frequency distribution of 26 systematically reviewed studies categorised as having high, low, or unclear risk of bias by each domain; the framework was adapted from Karalliu et al. [62].

The number of articles with low, unclear, or high risk of bias (of 26) for each domain is summarised in Figure 2. One of the ten domains, masking, was removed from Figure 2 because it was not applicable in most studies, except for our two interventional studies [38,39], in which there was no mention of masking (i.e., high risk of bias). The main factors leading to a high risk of bias included non-random, small, convenient sampling (35% of studies), and the lack of information regarding potential variables that could lead to bias (15.4%). In addition, in 61.5% of the studies, authors did not report how the sample size was calculated; therefore, the risk of bias regarding the study power was unclear.

## 4. Discussion

Although reproductive failure is widely recognised as one of the main concerns in pig production [2,7,17,42], our systematic review found a paucity of peer-reviewed studies focussed on reproductive failure in smallholder pig farms across East and Southeast Asia. As a result, veterinarians and farmers are often forced to rely on guidelines and recommendations derived from research conducted in intensive farming settings, either within the region or globally. While studies from intensive farms can offer valuable insights into general farm management, health, and production metrics, they may fall short of addressing the unique challenges faced by smallholder farms, such as relatively poor biosecurity, inadequate breeding stocks, limited access to veterinary services, and nutritional constraints [28,31].

Our review revealed that stillbirth, mummification, weak-born piglets, and late-term abortion are the most frequently reported types of reproductive failure in smallholder pig farms across East and Southeast Asia [44,45,46]. Diagnosing the causes of these reproductive failures remains inherently complex and challenging, as many failures exhibit overlapping clinical presentations, complicating the differentiation of aetiologic agents without in-depth diagnostic investigations [7]. Addressing these challenges requires an integrated approach, incorporating advanced laboratory diagnostics, systematic data collection, and interdisciplinary research collaborations, to elucidate the underlying mechanisms driving reproductive failure in pigs [6,7,28]. Notably, only three studies included in this review applied laboratory diagnostics to confirm the aetiologies of reproductive failure outbreaks in sow herds in Japan, Taiwan, and Vietnam [44,45,46]. These studies reported PCV-2 and coinfections with PCV-2 and PRRSV as the primary causative agents for the reported outbreaks. Given the high prevalence of these pathogens in intensive farming across the region [63,64], future epidemiological studies should specifically focus on smallholder farms to elucidate the infection dynamics within these settings. Such efforts are crucial for developing targeted prevention and control strategies that address the unique challenges faced by smallholder production systems.

While parasitic diseases are recognised as important contributors to reproductive failure in pigs [65], we found limited research attention given to parasitic causes, indicating a critical gap that future studies should address.

In addition to infectious agents, reproductive failure in pigs is influenced by a range of non-infectious factors [66]. Among these factors, our review highlighted extreme climate conditions (e.g., heat stress), inadequate nutrition, improper housing, and suboptimal boar management as important factors reducing reproductive performances in East and Southeast Asian smallholder farms [32,47,49,52]. Heat stress emerged as one of the main contributors to reduced fertility in breeding pigs [10,38,67,68]. The hot, humid summers typical of Southeast Asia and the extreme winter conditions in parts of East Asia exacerbate these stressors [13]. Many large-scale commercial farms in the region have proactively adopted technologies such as evaporative cooling and air conditioning to mitigate heat stress [10]. In contrast, most smallholder farmers lack access to the financial and technical resources required to implement such solutions. Interventions should, therefore, prioritise affordable, climate-adaptive technologies, such as improved housing designs and ventilation systems that are accessible to smallholders.

Our analysis also indicated a slight increase in the number of studies focused on smallholder pig farms after 2015, with most investigations centring on the identification and seroprevalence of infectious diseases. This slight surge in research activity could be attributed to the recurring outbreaks of economically important viral diseases. For instance, the devastating porcine epidemic diarrhoea virus (PEDV) outbreaks in late 2013 severely impacted countries such as South Korea, Taiwan, and Japan, with mortality rates reaching up to 100% in suckling piglets [69,70]. Similarly, the emergence of African swine fever (ASF) in China in 2018 triggered a regional crisis, rapidly spreading to neighbouring countries [14,15,25]. The African swine fever (ASF), characterised by high mortality rates and severe economic consequences [71,72], has underscored the urgent need for research, surveillance, and biosecurity interventions. Strengthening regional disease monitoring systems and fostering cross-border collaborations are essential to mitigating the impact of transboundary diseases and enhancing the resilience of the pig industry.

Geographically, research activities were concentrated in Vietnam, China, and Thailand, which collectively accounted for more than half of the studies included in our review. In contrast, no relevant studies were identified from Brunei, Singapore, Malaysia, Myanmar, Macau, South Korea, North Korea, or Mongolia. This apparent gap likely reflects variations in pig farming practices and pork consumption shaped by cultural, religious, and political factors. For example, Singapore ceased domestic pig farming in the 1980s, relying on imported frozen pork instead [73], while Brunei has no pig farming due to religious and cultural prohibitions [66]. In Malaysia, a predominantly Muslim country, the swine industry primarily supplies to the ethnic Chinese population [66]. Regional clustering of pig production is evident in Asia, with a major concentration in countries such as China, South Korea, Japan, Vietnam, the Philippines, and Thailand [74]. The lack of research from some countries highlights the need for a more inclusive approach to studying pig reproductive failures and production dynamics across the region. Future studies should aim to fill these geographical gaps by investigating pig production systems in under-represented areas, particularly in regions where smallholder pig farming persists as a critical livelihood strategy.

While we conducted a systematic and transparent review of the relevant literature, our synthesis has some limitations that should be acknowledged. First, our review was restricted to peer-reviewed journal articles written in English and Chinese due to limited accessibility, lack of standardisation, and potential language barriers associated with the grey literature. These restrictions may have excluded relevant studies, particularly from key pig-producing countries such as Myanmar, Cambodia, Laos, South Korea, and the Philippines, which were under-represented in our review. Second, as indicated in our quality assessment of the studies included, there was a substantial gap in reporting study design details. Many studies failed to conduct or report formal sample size calculations, which could likely reduce their statistical power in identifying significant risk factors. Moreover, a lack of clear documentation of sampling strategies was notable. Based on the available information, we inferred that many studies relied on convenience sampling, which may compromise both the internal validity (accuracy of findings within the study population) and external validity (generalizability of findings to broader target populations). Finally, a meta-analysis could not be conducted due to the lack of quantitative data and the heterogeneity in study designs and reporting practices among the included studies.

## 5. Conclusions

Our systematic review highlighted stillbirth, mummification, late-term abortion, and weak-born piglets as the predominant reproductive failures in smallholder pig farms across East and Southeast Asia, with PRRSV and PCV-2 identified as the most frequently cited viral pathogens associated with these failures. However, significant gaps were evident in most reviewed studies, particularly a lack of robust laboratory confirmation and a reliance on clinical and epidemiological observations. Addressing these gaps requires appropriate capacity building through investments in diagnostic infrastructure and the establishment of proper data collection frameworks to accurately identify and understand the causes of reproductive failure. Future research should focus specifically on reproductive failure in smallholder farms and adopt a comprehensive approach that incorporates infectious, environmental, and socioeconomic factors to ensure the development of relevant and practical solutions. Additionally, promoting affordable, climate-adaptive technologies, such as improved housing and cooling systems, can help mitigate the impact of extreme weather conditions (e.g., heat stress) on reproductive performance. These efforts would further support local stakeholders in devising targeted and cost-effective management strategies to enhance reproductive efficiency and productivity in smallholder pig farms, thereby ensuring food security and sustaining livelihoods across the region.

## Figures and Tables

**Table 1:** Domains and terms used to search the five databases.

Domain	Search Terms
Animal	Swine OR pig OR sow OR gilt OR porcine OR boar
Problem	Reproduct* OR fertility OR infertility
Production System	Smallholder OR extensive OR “free rang*” OR backyard OR “small scale” OR scavenging OR “semi-intensive” OR “family farm*”
Location	“Southeast Asia” OR “South-eastern Asia” OR Myanmar OR Cambodia OR Indonesia OR “Lao PDR” OR Malaysia OR Philippines OR Thailand OR Vietnam OR Timor Leste OR Singapore OR Brunei OR “East Asia” OR China OR “Hong Kong” OR Japan OR Macau OR “South Korea” OR “North Korea” OR Taiwan OR Mongolia
Final string	#Animal AND #Problem AND #Production system AND #Location

**Table 2:** Information extracted from selected studies on pig reproductive failure in East and Southeast Asia.

Bibliographic information	First author’s name; Title of the article; Journal name; Publication year; DOIs/URLs; Authors’ stated objective(s).
Study setting	Country where the study was conducted; Study location (district/province, cities); Study period; Study commencement year; Study type (descriptive survey, cross-sectional, case–control, cohort, or interventional/clinical trial); Sampling method; Sample size.
Pig population and operation	Pig production operation (breeder, farrow-to-finish, or finisher); Pig category (piglet, sow, gilt, boar, weaner, grower); Pig breed(s).
Reproductive failure	Reproductive failures, such as abortion, infertility (non-pregnant, anoestrous, genital defects), low farrowing rate, pseudopregnancy, stillbirth, premature litters, weak piglets; Infectious diseases causing reproductive failure; Risk factor(s) for reproductive failure other than infectious diseases/pathogens (including environment, nutrition, and genetics).

## Data Availability

All data related to this study are freely available in the manuscript and Supplementary Materials.

## Supplementary Materials

The following supporting information can be downloaded at https://www.mdpi.com/article/10.3390/ani15091226/s1, Table S1. A complete list of studies, including all information collected for this systematic review.

## Abbreviations

The following abbreviations are used in this manuscript:

AI	Artificial Insemination
ASFV	African Swine Fever Virus
CSFV	Classical Swine Fever Virus
FMD	Foot-and-Mouth Disease
IAV	Influenza A Virus
JEV	Japanese Encephalitis Virus
PCV-2	Porcine Circovirus Type 2
PEDV	Porcine Epidemic Diarrhoea Virus
PPV	Porcine Parvovirus
PRISMA	Preferred Reporting Items for Systematic Reviews and Meta-Analyses
PRRSV	Porcine Reproductive and Respiratory Syndrome Virus
PRV	Pseudorabies Virus

## Appendix A

**Table A1:** Domains and terms used to search the five databases (in English and Chinese).

Domain	Search Terms
Animal	Swine (家豬) OR pig (豬) OR sow (母豬) OR gilt (豬女) OR porcine (豬) OR boar (公豬)
Problem	Reproduct* (生殖) OR fertility (生育) OR infertility (不孕不育)
Production System	Smallholder(小農) OR extensive (粗放) OR “free rang*” (散養) OR backyard (後院) OR “small scale” (小規模) OR scavenging (放養) OR “semi-intensive” (半集約) OR “family farm*” (家庭農場)
Location	“Southeast Asia” (東南亞) OR “South-eastern Asia” OR Myanmar (緬甸) OR Cambodia (柬埔寨) OR Indonesia (印尼) OR “Lao PDR”(老挝人民共國) OR Malaysia (馬來西亞) OR Philippines (菲律賓) OR Thailand (泰國) OR Vietnam (越南) OR Timor-Leste (東帝汶) OR Singapore (新加玻) OR Brunei (汶萊) OR “East Asia” (東亞) OR China (中國) OR “Hong Kong”(香港) OR Japan (日本) OR Macau (澳門) OR “South Korea”(南韓) OR “North Korea”(北韓) OR Taiwan (台灣) OR Mongolia (蒙古)
Final String	#animal AND #problem AND #production system AND #location

**Table A2:** Distribution of 26 reviewed studies on pig reproductive failure in East and Southeast Asian smallholder pig farms by journal.

Journal Name	Journal Type	Number of Papers (%)	Publication Year	Full-Text Language
*Asian Agricultural Research*	Regional	1(4)	2021	English
*BMC Veterinary Research*	International	1(4)	2020	English
*China National Knowledge Infrastructure*	Regional	1(4)	2019	Chinese
*Canadian Veterinary Journal*	International	1(4)	2018	English
*Journal of Comparative Pathology*	International	1(4)	2016	English
*Journal of the Indonesian Tropical Animal Agriculture*	Regional	1(4)	2022	English
*Journal of Veterinary Medical Science*	International	1(4)	2005	English
*New Zealand Veterinary Journal*	International	1(4)	2014	English
*Pathogens*	International	1(4)	2021	English
*Philippine Journal of Veterinary and Animal Sciences*	Regional	1(4)	2015	English
*Preventive Veterinary Medicine*	International	5(19)	2002–2021	English
*Research in Veterinary Science*	International	1(4)	2020	English
*Thai Journal of Veterinary Medicine*	Regional	1(4)	2022	English
*Transboundary and Emerging Diseases*	International	1(4)	2017	English
*Tropical Animal Health and Production*	International	4(15)	2010–2018	English
*Veterinary Microbiology*	International	1(4)	2006	English
*Veterinary Parasitology*	International	1(4)	2021	English
*Veterinary Sciences*	International	1(4)	2022	English
*Chinese Journal of Animal and Veterinary Science*	Regional	1(4)	2013	Chinese
